# **Imaging-detected extranodal extension in head and neck cancer: clinical implications, diagnostic criteria**, **and the potential of photon-counting detector CT**

**DOI:** 10.1007/s11604-025-01894-3

**Published:** 2025-10-16

**Authors:** Hirofumi Kuno, Takashi Hiyama, Tomoaki Sasaki, Shingo Sakashita, Ryutaro Onaga, Toshifumi Tomioka, Yoshihisa Muramatsu, Naruomi Akino, Hiroki Taguchi, Kotaro Sekiya, Tatsushi Kobayashi

**Affiliations:** 1 Department of Diagnostic Radiology, National Cancer Center Hospital East, 6-5-1, Kashiwanoha, Kashiwa, Chiba 277-8577 Japan; 2 Department of Pathology and Clinical Laboratories, Division of Pathology, National Cancer Center Hospital East/National Cancer Center Exploratory Oncology Research & Clinical Trial Center, Kashiwa, Japan; 3 Department of Head and Neck Medical Oncology, National Cancer Center Hospital East, Kashiwa, Japan; 4 Department of Otolaryngology and Head and Neck Surgery, Jichi Medical University, Tochigi, Japan; 5 Department of Head and Neck Surgery, National Cancer Center Hospital East, Kashiwa, Japan; 6 Canon Medical Systems Corporation, Otawara, Japan

**Keywords:** Extranodal extension, Head and neck cancer, Photon-counting CT (PCCT/PCD-CT), Spectral imaging, Lymph node metastasis, TNM staging

## Abstract

Imaging-detected extranodal extension (iENE) is increasingly recognized as an objective adverse prognostic factor in head and neck cancer. The newly published 9th edition of the UICC/AJCC TNM Classification now incorporates iENE into the nodal (N) categories for nasopharyngeal carcinoma, HPV-associated oropharyngeal carcinoma, and salivary gland carcinoma, typically upstaging patients to higher N categories, and designates imaging findings as an accepted standard for the clinical ENE (cENE) as the N3b category in all other head and neck subsites. To establish diagnostic criteria for iENE, the Head and Neck Cancer International Group (HNCIG) has proposed a four-tier grading system (2024): Grade 0 (iENE-negative), Grade 1 (perinodal fat extension), Grade 2 (coalescent lymph nodes), and Grade 3 (invasion of adjacent structures). Each grade carries distinct clinical implications, ranging from treatment de-escalation for Grade 0 to consideration of unresectable disease for Grade 3. Recent advances in ultra-high-resolution imaging, particularly photon-counting detector CT (PCD-CT) with 1024 × 1024 matrix in super-high-resolution (SHR) mode, may offer the spatial details necessary to more reliably detect these subtle findings compared with conventional imaging. Through direct comparisons with histopathological correlations, we demonstrate the potential of high-resolution imaging in detecting iENE. PCD-CT, with its simultaneous spectral imaging capabilities—including virtual monochromatic imaging—enhances soft tissue contrast for detecting subtle ENEs. In addition, material decomposition maps aid in the precise evaluation of tumor–tissue interfaces. This comprehensive review is an educational resource for radiologists applying the HNCIG consensus criteria including the newly adopted TNM staging requirements. While advanced imaging technologies such as PCD-CT show promising potential for enhanced tissue characterization, further validation studies are needed to establish their clinical utility in routine iENE assessment.

**Supplementary Information:**

The online version contains supplementary material available at 10.1007/s11604-025-01894-3.

## Introduction

Extranodal extension (ENE) represents one of the critical adverse prognostic factors in head and neck squamous cell carcinoma (HNSCC). ENE is a pathologic condition in which metastatic tumor cells invade surrounding tissues through the lymph node capsule [1–6] and significantly worsen patient outcomes by decreasing local control rates and increasing the risk of distant metastasis [1, 7, 8]. The clinical importance of ENE has been recognized for decades, leading to its formal incorporation into the UICC/AJCC 8th edition staging system in 2018 [8]. According to these guidelines, ENE automatically upstages patients to higher N categories in most head and neck cancer subsites, regardless of the number or size of involved lymph nodes. This reflects the profound impact of ENE on both prognosis and treatment planning.

Traditionally, ENE has been assessed through two primary methods: clinical examination and histopathological evaluation. Clinical ENE (cENE) is typically diagnosed only when overt signs, such as nodal fixation, are observed. In contrast, pathologic ENE requires surgical resection for a definitive diagnosis. However, these methods have limitations—particularly in the context of a modern multidisciplinary approach, where many patients receive nonsurgical treatment—raising concerns about patient stage migration [7–10].

To address this gap, imaging-detected ENE (iENE) has emerged as an objective, non-invasive alternative that can be applied across all treatment modalities [7, 9, 11–13]. Recognizing the need for standardization, the Head and Neck Cancer International Group (HNCIG) established consensus-based diagnostic criteria and a systematic grading framework for iENE assessment [11]. Building on these guidelines, the upcoming UICC 9th edition recognizes iENE as an independent prognostic factor [14–16]. The UICC/AJCC TNM Classification 9th edition now formally incorporates iENE into the nodal (N) categories for nasopharyngeal carcinoma, HPV-associated oropharyngeal carcinoma, and salivary gland carcinoma, typically upstaging patients to higher N categories. In addition, for all other head and neck subsites, the 9th edition designates imaging findings as the accepted standard for clinical ENE (cENE) determination, classifying such cases as the N3b category [15].

Simultaneously, advancements in imaging technology—particularly photon-counting detector CT (PCD-CT), also referred to as photon-counting CT (PCCT), with 1024-matrix resolution—enable more detailed visualization of anatomical structures compared to conventional imaging techniques [17–19]. These technological innovations, combined with standardized diagnostic criteria, offer the potential for more accurate and reproducible iENE assessments.

This review aims to provide radiologists with a comprehensive understanding of the HNCIG consensus criteria for iENE diagnosis, demonstrate the added value of high-resolution imaging techniques, including PCD-CT, through correlation with pathological findings, and offer practical guidance for integrating these criteria into clinical practice to improve patient care in the era of precision oncology.

## ENE in head and neck cancer: definitions and current limitations

### Definition and terminology

Three terms are used to describe ENE in head and neck cancers: pathological ENE (pENE), cENE, and iENE. The term “i = radiological imaging-detected” ENE is the standardized terminology adopted by both the HNCIG consensus guidelines and the UICC/AJCC TNM Classification 9th edition [11, 15], avoiding potential confusion with “r = recurrence” in TNM nomenclature.

Early-stage ENE can be detected only through surgical pathology (pENE) [5, 12, 20]. As ENE progresses, it becomes visible on radiological imaging (iENE). Further progression may lead to skin infiltration or invasion of adjacent structures, causing adhesion or cranial nerve palsy, and ultimately resulting in clinically evident ENE (cENE) [8]. Not all cases of pENE, however, are detectable on imaging due to the inherent differences in assessment—macroscopic radiological examination versus microscopic histopathological evaluation [11, 21, 22].

### pENE and current limitations

pENE is defined as the histological detection of tumor spread beyond the lymph node capsule and is a strong predictor of poor prognosis in surgically treated HNSCC [2, 3]. It remains a standard indication for adjuvant chemoradiation following definitive surgery. pENE is further classified into minor (≤ 2 mm), major (> 2 mm), and soft tissue metastasis (STM), the latter referring to tumor deposits without evidence of residual node or nodal architecture [8, 20].

Despite its established role in clinical decision-making, pENE assessment has several limitations. Sampling errors can lead to false negatives, as microscopic extension may be missed in a single cross-sectional sample [20, 23, 24]. Moreover, pENE assessment is restricted to surgical cases, excluding patients treated nonsurgically. Diagnostic variability across institutions—due to the lack of standardized histological criteria—also contributes to inconsistent reporting [20]. Recent studies have questioned the prognostic value of minor pENE [23, 25, 26]. A large multicenter study demonstrated that adjuvant chemotherapy offered no survival benefit for patients with minor ENE but significantly improved outcomes in those with major pENE [26]. These findings suggest that current treatment paradigms may lead to overtreatment of minor pENE cases and indicate the need for more refined, universally applicable prognostic markers—especially for patients who do not undergo surgery.

### cENE and current limitations

cENE refers to a visible or palpable tumor that extends beyond the lymph node capsule and is detectable during physical examination [8, 27]. It represents a poor prognostic indicator in head and neck squamous cell carcinoma (HNSCC), and its presence classifies a patient as N3b in most subsites, according to the AJCC 8th edition. However, cENE assessment is challenging and subjective, with significant discrepancies between clinical findings and radiological evidence; it has been reported that fewer than half of patients with imaging-detected ENE (iENE) present with clinical signs of cENE [10, 28]. This suboptimal detection may deprive patients undergoing nonsurgical treatment of accurate prognostic information, potentially compromising treatment optimization.

While the 8th edition of the AJCC considered radiological evidence insufficient for cENE diagnosis, the 9th edition acknowledged the expanding role of imaging [8, 15]. Although maintaining the clinical definition of extranodal extension, the 9th edition adds that “imaging is becoming a standard method of detecting unequivocal extranodal extension” [15], facilitating more standardized evaluation across all treatment modalities.

## iENE in head and neck cancer: HNCIG iENE grading consensus recommendations

iENE refers to the radiologically visible spread of tumors beyond the lymph node capsule. It has emerged as a critical area of investigation in head and neck cancer, with growing attention to standardization and improved diagnostic accuracy. Recent studies indicate that iENE is a key predictive factor in the pretreatment assessment of head and neck cancer, offering a non-invasive means of risk stratification and influencing both treatment planning and patient outcomes [7, 10, 24, 29–33]. Modern imaging techniques offer advantages such as multiplanar reconstruction in any orientation and high-resolution nodal evaluation with thin slices (< 1 mm). However, limitations remain—particularly the absence of standardized assessment criteria and high inter-observer variability—which have historically hindered the incorporation of iENE into clinical decision-making.

**Fig. 1:**
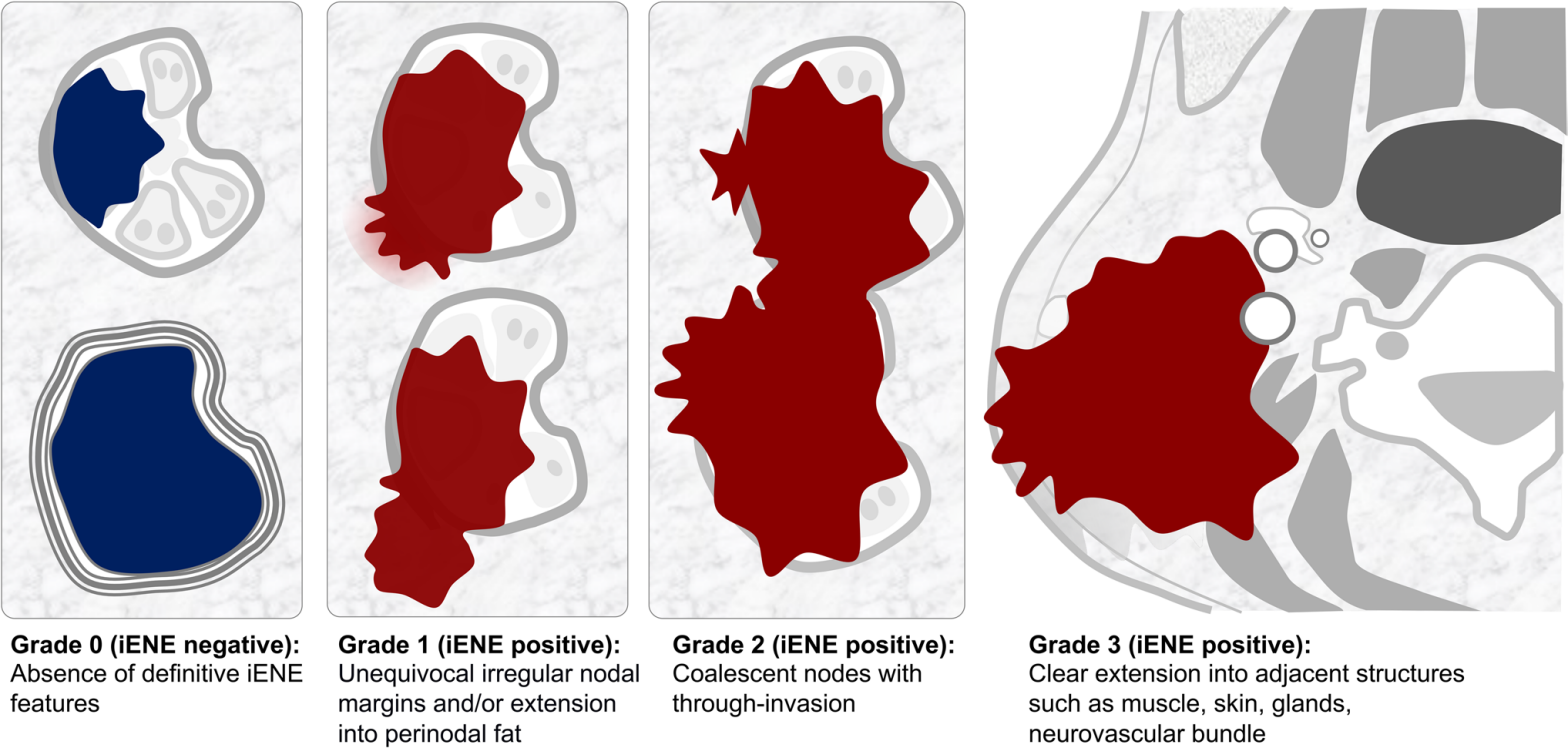
HNCIG iENE grading system. Head and Neck Cancer International Group (HNCIG) consensus classification for imaging-detected extranodal extension (iENE). The four-tier grading system categorizes iENE from Grade 0 (negative) to Grade 3 (extension into adjacent structures). Grade determination is based on the highest grade finding with certainty, and equivocal findings are considered “not applicable.” When criteria for multiple grades are met, the highest grade with definitive radiologic evidence is assigned

Recognizing the need for standardization, the HNCIG convened a global consensus panel in 2024, bringing together 21 member organizations. Through a five-round modified Delphi process involving 18 international radiology experts, the panel developed consensus guidelines on iENE terminology and diagnostic criteria [9, 11, 13]. The AOSNHNR-ASHNR-ESHNR Joint Task Force has subsequently provided additional refinements to these criteria [ref: https://ashnr.org/iene/] [34]. The agreed-upon radiologic features of iENE include indistinct nodal margins, conglomerate/matted/coalescent nodes, and extension into perinodal fat or adjacent structures. These criteria apply to both HPV-associated and non-HPV-associated cancers [11]. Notably, nodal necrosis and capsular thickening or enhancement were excluded as diagnostic features of iENE [11]. The panel also noted that diagnostic accuracy may be compromised if information about a recent core biopsy is unavailable. Based on these findings, a new four-tier iENE grading system was proposed **(**Fig. 1**)**: Grade 0 (iENE negative) with no radiological features of iENE; Grade 1: unequivocal irregular nodal margins and/or extension confined to perinodal fat; Grade 2: clear invasion through two or more inseparable adjoining lymph nodes (conglomerate/matted/coalescent nodes) with or without Grade 1 features; and Grade 3: clear extension into adjacent structures such as muscle, skin, glands, or neurovascular bundle with or without Grade 1 or 2 features [11].

Grading is assigned based on the highest unequivocal feature observed. Equivocal findings are categorized as “not applicable.” When criteria for multiple grades are met, the highest grade with definitive evidence is assigned [11, 34]. For instance, if there is definitive extension into perinodal fat (Grade 1) but only a possible muscle invasion (equivocal for Grade 3), the lesion is assigned Grade 1 rather than Grade 3. This structured approach supports standardized iENE assessment across institutions, enabling more consistent prognostic evaluation and improved treatment planning in head and neck cancer [9, 11, 13]. The detailed imaging criteria for each grade, integrating the HNCIG consensus recommendations with refinements from the AOSNHNR-ASHNR-ESHNR Joint Task Force, are summarized in Table 1 [11, 34].

**Table 1:** HNCIG consensus criteria for iENE grading: essential imaging findings and potential role of advanced techniques*

Grade	Essential imaging findings (conventional CT/MRI)	Potential advantages of high-resolution and spectral imaging
Grade 0 (iENE-negative)	Absence of unequivocal ENE: • Smooth, well-defined nodal margins • Equivocal features (e.g., central necrosis, capsular thickening/enhancement) without definitive extension into perinodal fat	May improve diagnostic confidence: • High resolution (1024 × 1024 matrix) may improve visualization of nodal margins and the perinodal fat plane, helping to more confidently rule out subtle extension
Grade 1 (iENE-positive)	Unequivocal perinodal fat extension: • Defined by projections or spikes into fat (irregular capsule alone is insufficient) • MRI: “Flare sign” (high signal on fat-suppressed T2WI) is highly specific *Caution: findings can be mimicked by post-treatment, inflammatory, or post-biopsy changes	May enhance detection of subtle fat infiltration: • Low-keV virtual monochromatic images (VMIs) may improve soft tissue contrast, enhancing the visibility of subtle perinodal stranding • Spectral fat maps may help confirm fat plane obliteration • Higher spatial resolution potentially improves visualization of subtle perinodal stranding
Grade 2 (iENE-positive)	Coalescent/matted nodes, requiring all three criteria: 1. Loss of intervening fat planes 2. Loss of convexity at the point of contact 3. Merging of nodal boundaries (loss of acute angle) *Caution: Must be distinguished from a single large necrotic/lobulated node (multiplanar imaging may be needed)	May improve differentiation of true coalescence: • High-resolution (1024 × 1024 matrix) potentially allows more detailed visualization of interfaces between adjacent nodes • May help distinguish true capsular disruption from anatomical proximity
Grade 3 (iENE-positive)	Clear extension into adjacent structures, defined by unequivocal invasion: • Muscle: Direct muscle infiltration (loss of fat plane alone is insufficient) • Vessels: Arterial encasement ≥ 270° (major arteries only); internal jugular vein obliteration or tumor thrombus (compression alone is insufficient) • Other structures: invasion into skin (with subcutaneous fat loss), gland parenchyma (not just displacement), or nerve (with denervation signs)	May improve characterization of tissue interfaces: • High-resolution imaging and iodine maps may better delineate tumor-adjacent structure interfaces • Iodine subtraction maps may help delineate the tumor-vessel interface • VMIs at low keV levels may enhance evaluation of muscle and soft tissue interfaces

^*^Criteria are based on the HNCIG consensus recommendations and further refined by the AOSNHNR-ASHNR-ESHNR Joint Task Force guidelines [11, 34]. High-resolution imaging includes ultra-high-resolution CT (UHR-CT) and photon-counting detector CT (PCD-CT) with 1024 × 1024-matrix capability

## Advanced CT imaging for iENE: high-resolution techniques and the potential role of PCD-CT

PCD-CT is an emerging imaging technology with the potential to significantly impact clinical practice [17, 18]. Compared to conventional energy-integrated detector CT (EID-CT), PCD-CT offers several advantages, including improved spatial resolution, reduced image noise, multi-energy spectral imaging capabilities, and reduced radiation exposure [17, 19, 35, 36]. Regarding spatial resolution improvements, conventional EID-CT commonly employs a 512 × 512 matrix reconstruction, termed normal resolution (NR) mode. Recent advances in high spatial resolution technologies [36–38], including PCD-CT, now enable super-high-resolution (SHR) mode using 1024 × 1024-matrix reconstruction with thinner slice acquisition, facilitating more detailed ENE assessment. The improvement in spatial resolution enhances the evaluation of lymph node margins and boundaries, enabling better visualization of subtle ENE features.

Simultaneously, PCD-CT can generate spectral images, such as virtual monochromatic images and iodine maps [17–19, 36]. In particular, low keV images in the virtual monochromatic imaging specifically enhance contrast between iodine-enhanced blood vessels, lymph node tissue, and surrounding fat tissue. These low keV images may facilitate the detection of subtle vascular infiltration and surrounding fat invasion, potentially offering clinical utility in ENE diagnosis. Both SHR mode and low keV images can potentially achieve noise reduction through deep learning reconstruction (DLR) techniques, resulting in improved image quality and enhanced contrast [39, 40].

In this review, the presented images were acquired from several systems to illustrate these technologies. To establish a baseline for comparison, “normal resolution” (NR) images were obtained in two ways: some were acquired directly on a conventional EID-CT scanner, and others were generated as “simulated NR” images (512 × 512 matrix with adaptive iterative dose reduction 3D [AIDR 3D] reconstruction) from an ultra-high-resolution CT (UHR-CT) scanner (Aquilion Precision; Canon Medical Systems, Otawara, Japan) [41]. The high-resolution images shown are super-high-resolution (SHR) images (1024 × 1024 matrix with deep learning reconstruction [DLR]), generated from a UHR-CT and a cadmium-zinc-telluride (CZT)-based PCD-CT system (TSX-501R; Canon Medical Systems, Otawara, Japan). The PCD-CT scanner provides image reconstruction at a thin slice thickness of 0.2 mm, and the DLR used for the PCD-CT images was adapted specifically for its photon-counting data. The precise parameters for each figure are detailed in the respective legends. Standardization of imaging protocols and validation through dedicated research studies remain necessary to establish the clinical utility of these capabilities in iENE assessment.

In the following sections, we will use clinical cases to illustrate the imaging findings for each of the four iENE grades. The specific diagnostic criteria for each grade, including the potential role of PCD-CT, are detailed in Table 1 for reference.

## iENE-negative: Grade 0

### Definition

iENE Grade 0 is defined as the absence of definitive radiologic signs of ENE when ENE findings are either uncertain or not present **(**Fig. 1**)**. It is important to note that iENE is a newly recognized, independent prognostic factor and is not equivalent to pENE. As such, conditions corresponding to minor pENE—which cannot be reliably identified on imaging—are classified as iENE-negative (Grade 0). According to the HNCIG iENE consensus, certain criteria previously used in some studies comparing imaging diagnostic performance with the gold standard of pENE, such as tumor necrosis and capsular thickening, are now regarded as uncertain indicators for iENE diagnosis [11]. Grade 0 includes not only lymph nodes with smooth, well-defined margins but also cases with equivocal features in which definitive ENE cannot be established. This distinction holds clinical importance, as irregular nodal contours may reflect capsular thickening or inflammation rather than true ENE. This interpretation aligns with the UICC 9th edition’s adoption of “unequivocal iENE,” which sets a standard whereby uncertain findings are not classified as positive.

### Clinical significance

This definition carries significant clinical implications for the management of head and neck cancers. A confirmed iENE-negative (Grade 0) status is now an explicit eligibility criterion for transoral robotic surgery (TORS) in HPV-associated oropharyngeal carcinoma, as outlined in the 2025 ASCO guidelines. As such, it serves as a critical factor in therapeutic decision-making [42–44]. Retrospective analyses have consistently demonstrated that iENE-positive status is associated with inferior survival outcomes in laryngeal and hypopharyngeal cancers treated with definitive chemoradiotherapy [45], as well as unfavorable treatment response in locally advanced HNSCC managed with induction chemotherapy followed by chemoradiotherapy [46]. These findings emphasize the importance of accurate iENE assessment for risk stratification and treatment planning.

### Imaging findings for iENE Grade 0 and advantages of PCD-CT

**Fig. 2:**
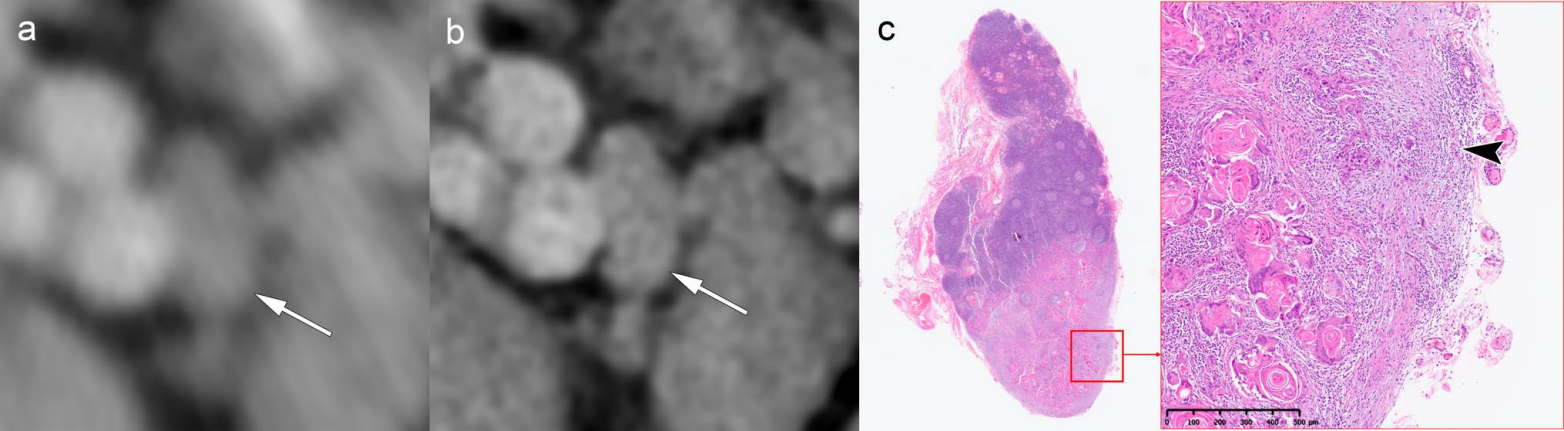
Imaging limitations in detecting minor pathologic ENE in clinically node-negative disease. A 46-year-old man with tongue squamous cell carcinoma, clinically staged as cN0. **a** A simulated normal resolution (NR) CT image fails to identify nodal metastasis (arrow). **b** A super-high-resolution (SHR) CT image from the same UHR-CT shows improved detail but no definitive nodal abnormality (arrow). **c** Histopathology from prophylactic neck dissection reveals lymph node metastasis with minor pENE (< 2 mm, arrowhead), resulting in upstaging from cN0 to pN3b. Imaging parameters: **a** ultra-high-resolution CT (UHR-CT); simulated NR mode, 512 × 512 matrix, 1-mm slice thickness, Adaptive Iterative Dose Reduction 3D (AIDR 3D). **b** UHR-CT; SHR mode, 1024 × 1024 matrix, 1-mm slice thickness, deep learning reconstruction (DLR)

Achieving diagnostic confidence in assigning iENE Grade 0 requires careful interpretation of specific imaging findings (Table 1). Figure 2 demonstrates a case where microscopic (< 2 mm) pENE, confirmed histologically, remained undetectable even with 1024-matrix acquisition. Such cases falling below the imaging detection threshold are appropriately classified as iENE Grade 0, reflecting current imaging limitations.

**Fig. 3:**
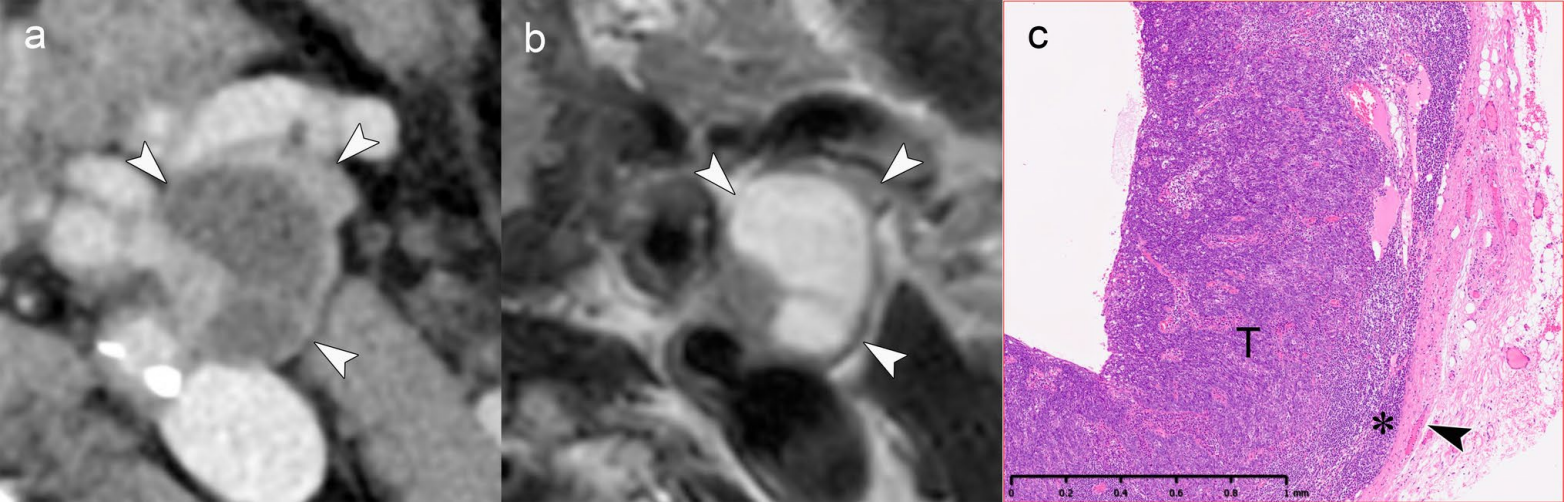
iENE Grade 0 with central necrosis and capsular thickening. An 83-year-old man with p16-positive oropharyngeal carcinoma (T2N1M0). **a** Ultra-high-resolution CT shows a lymph node with smooth margins despite central necrosis (arrowheads). **b** T2-weighted MRI demonstrates central necrosis and capsular thickening, but preserved perinodal fat planes (arrowheads). **c** Histopathology confirms preserved lymphoid tissue (*) between tumor (T) and thickened capsule (arrowhead), validating the iENE Grade 0 classification (pENE negative). The patient underwent transoral robotic surgery based on the confirmed iENE-negative status. Imaging parameters: **a** UHR-CT; SHR mode, 1024 × 1024 matrix, 1-mm slice thickness, DLR. **b** 3 T MRI; axial T2-weighted sequence, 3 mm slice thickness

**Fig. 4:**
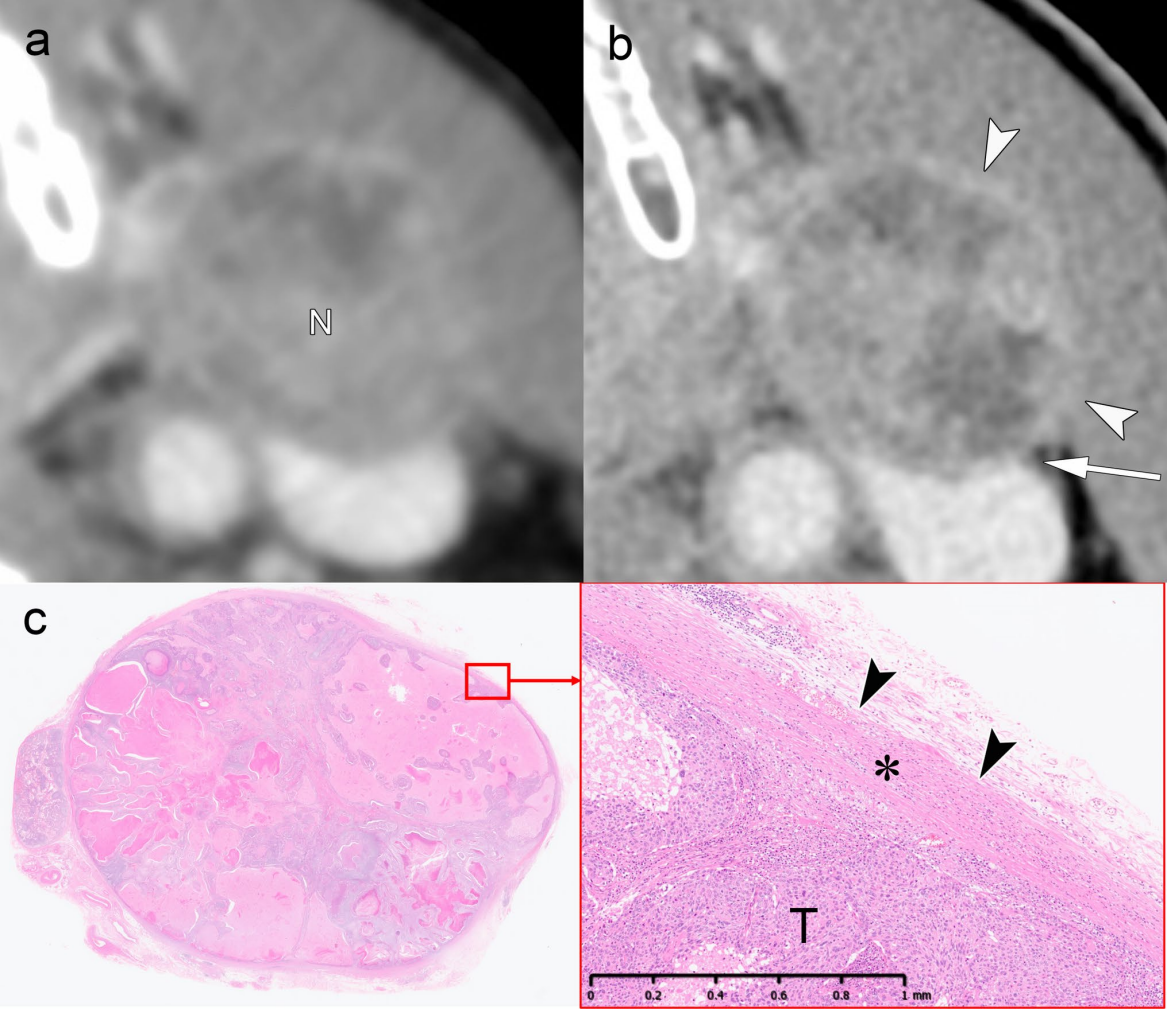
iENE Grade 0 with capsular thickening in an 81-year-old man with hypopharyngeal squamous cell carcinoma. **a** A simulated normal resolution (NR) CT image shows lymph node metastasis (N) with indistinct margins. **b** A super-high-resolution (SHR) CT image from the same UHR-CT provides more precise margin delineation (arrowheads) and shows internal jugular vein compression (arrow) without definitive irregularity or extension into the surrounding fat. **c** Histopathological examination demonstrates thickening of the fibrous capsule (*) without evidence of ENE (arrowheads). *T* Tumor. Imaging parameters: **a** UHR-CT; simulated NR mode, 512 × 512 matrix, 1 mm slice thickness, Adaptive Iterative Dose Reduction 3D (AIDR 3D). **b** UHR-CT; SHR mode, 1024 × 1024 matrix, 1 mm slice thickness, DLR

**Fig. 5:**
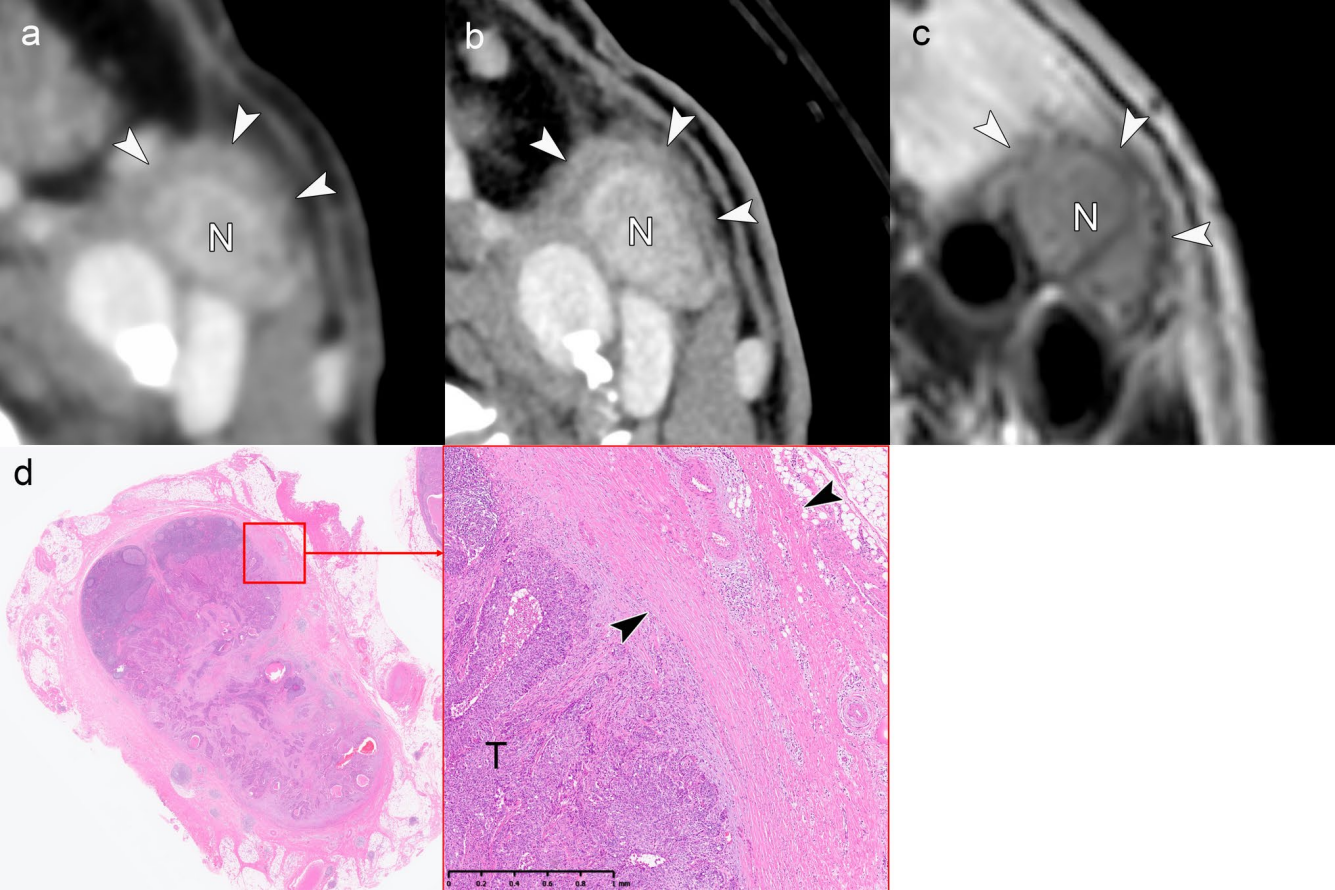
Equivocal iENE findings classified as Grade 0: irregular nodal capsule without extranodal extension. A 75 year-old man with hypopharyngeal squamous cell carcinoma and level II lymph node metastasis. **a** A simulated normal resolution (NR) CT image demonstrates lymph node metastasis with irregular margins (arrowheads), raising concern for possible extranodal extension. **b** A super-high-resolution (SHR) CT image from the same UHR-CT better delineates the irregular but intact capsule as a hypodense band (arrowheads), distinguishing it from the enhancing metastatic component (N). **c** T2-weighted MRI (3 mm slice thickness) visualizes the preserved capsular structure as a low-signal intensity band with irregular contour (arrowheads), confirming capsular integrity. **d** Histopathological examination reveals a thickened fibrous capsule (arrowheads) without evidence of ENE, validating the Grade 0 classification despite irregular imaging appearance. *T* Tumor, *N* Lymph node. Imaging parameters: **a** UHR-CT; simulated NR mode, 512 × 512 matrix, 1-mm slice thickness, AIDR 3D. **b** UHR-CT; SHR mode, 1024 × 1024 matrix, 1 mm slice thickness, DLR. **c** 3 T MRI; axial T2-weighted sequence, 3 mm slice thickness

Figures 3 and 4 present cases with indeterminate findings—central necrosis and capsular thickening, respectively—that the HNCIG consensus classifies as iENE Grade 0. In these cases, high-resolution imaging enhanced visualization of nodal margins and perinodal fat planes, facilitating more confident exclusion of unequivocal ENE compared to conventional detectors. Figure 5 illustrates how T2-weighted MRI provides additional diagnostic value by demonstrating preserved capsular architecture as a hypointense rim, differentiating an irregular but intact capsule from true extranodal extension. While early clinical experience with these advanced imaging techniques is promising, prospective validation studies are needed to establish their actual diagnostic impact on inter-reader agreement and clinical outcomes.

## iENE-positive: Grade 1

### Definition

iENE Grade 1 is defined by clearly irregular or ill-defined nodal margins and/or extension into the perinodal fat that is confined to the perinodal adipose tissue **(**Fig. 1**)** [11]. The AOSNHNR-ASHNR-ESHNR Joint Task Force emphasizes that irregular or indistinct capsule alone is insufficient; these must be accompanied by projections or spikes into the perinodal fat [34]. This grade represents the earliest radiologically detectable form of ENE and serves as a key threshold separating iENE-negative from iENE-positive disease. Diagnosis requires unequivocal imaging findings that distinguish it from the uncertain features characterizing Grade 0 status [11, 34].

### Clinical significance

This precise radiologic distinction holds important clinical implications for the management of contemporary head and neck cancers. The ICON-N study by Huang et al. (2025) identified Grade 1 iENE as a critical prognostic factor and provided the foundation for its inclusion in the 9th edition of the UICC staging system for HPV-associated oropharyngeal carcinoma [14]. In this international multicenter study involving 2,053 patients, iENE emerged as the strongest prognostic nodal feature, with an adjusted hazard ratio of 2.43 (95% CI, 1.96–3.03). This study also showed that reclassifying iENE-positive N1 disease one stage higher resulted in improved prognostic stratification, with a 5-year overall survival rate of 71% for iENE-positive cases versus 87% for iENE-negative cases. This evidence-based approach has now been formally adopted in the UICC 9th edition N classification for HPV-associated oropharyngeal carcinoma [14, 15].

The prognostic value of iENE Grade 1 extends beyond HPV-associated oropharyngeal carcinoma and has been consistently validated across multiple head and neck cancer subtypes and treatment modalities. Several retrospective studies have demonstrated that iENE-positive disease is associated with reduced locoregional control, higher rates of distant metastasis, and poorer overall survival outcomes regardless of the primary treatment approach [24, 30–33, 45, 47]. Similarly, Onaga et al. (2024) found that iENE Grade 1 predicted a poorer treatment response and prognosis in patients with locally advanced HNSCC treated with induction chemotherapy followed by chemoradiotherapy [46]. These findings underscore the importance of accurate iENE assessment in identifying patients who may benefit from treatment intensification due to adverse prognostic features.

### Imaging findings for iENE Grade 1 and advantages of PCD-CT

**Fig. 6:**
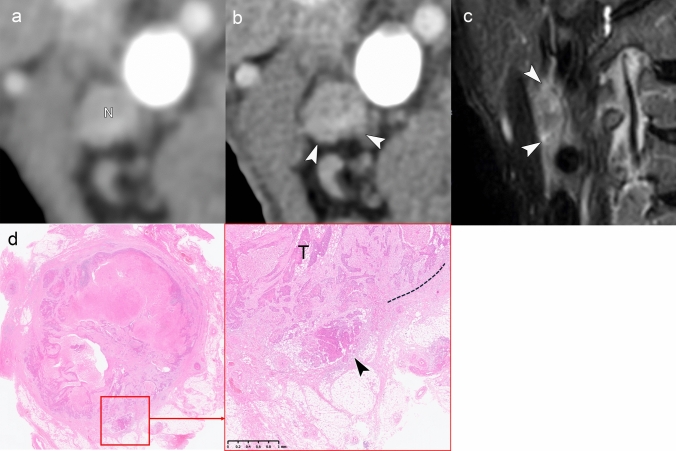
iENE Grade 1 in a 73 year-old man with buccal mucosal squamous cell carcinoma. **a** A simulated normal resolution (NR) CT image shows limited nodal detail. **b** A super-high-resolution (SHR) CT image from the same UHR-CT clearly demonstrates irregular margins with projections/spikes extending into perinodal fat extension (arrowheads). **c** MRI short tau inversion recovery sequence (coronal, 3 mm slice thickness) reveals high signal intensity around the lymph node (“flare sign”), indicating ENE. **d** Histopathological examination confirms metastatic tumor (T) extension through the lymph node capsule into surrounding connective tissue (arrowheads), consistent with minor pathologic ENE. T: tumor; N: lymph node. Imaging parameters: **a** UHR-CT; simulated NR mode, 512 × 512 matrix, 1 mm slice thickness, AIDR 3D. **b** UHR-CT; SHR mode, 1024 × 1024 matrix, 1 mm slice thickness, DLR. **c** 3 T MRI; coronal STIR sequence, 3 mm slice thickness

**Fig. 7:**
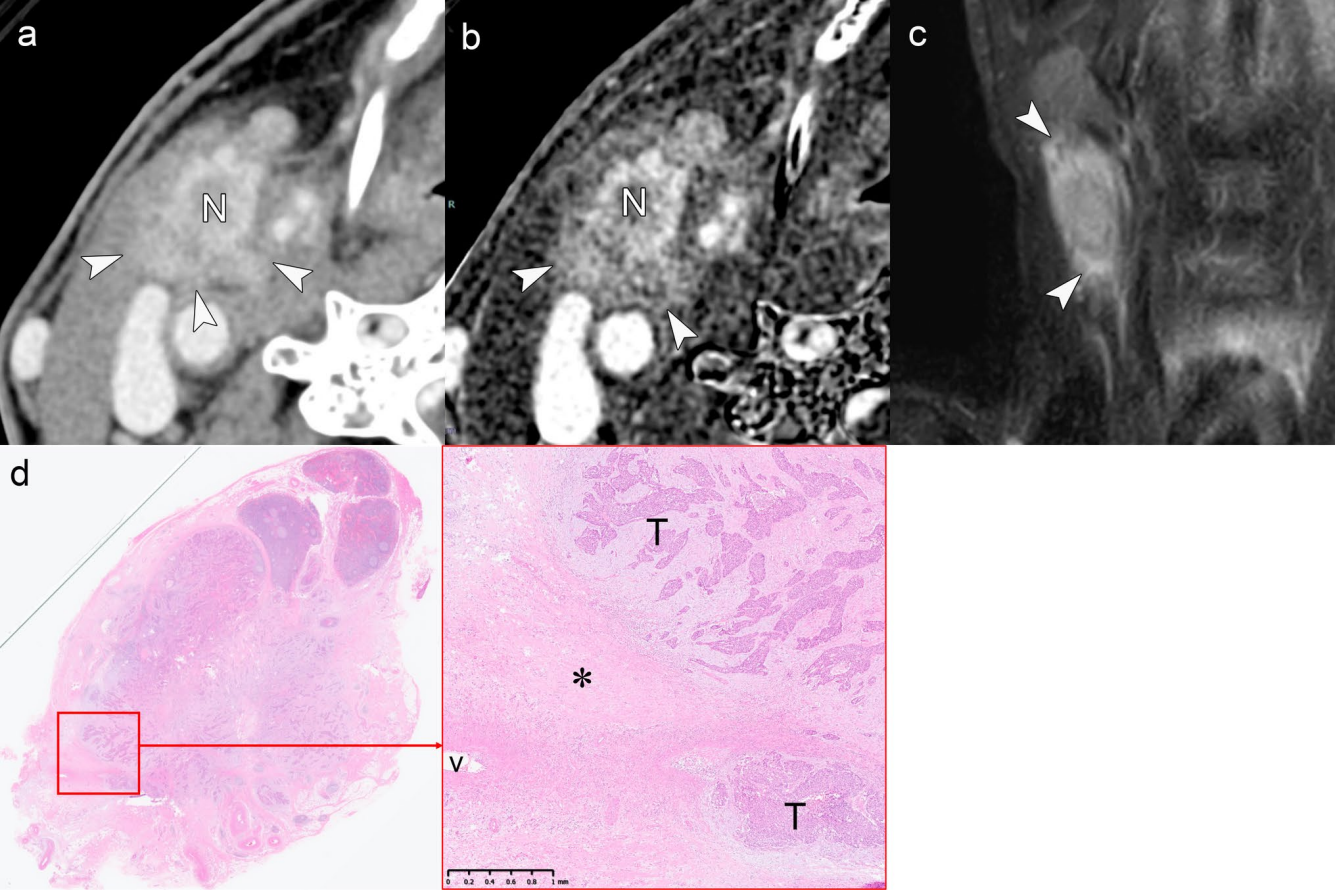
iENE Grade 1 in a 75 year-old man with hypopharyngeal squamous cell carcinoma. **a** Ultra-high-resolution CT improves margin delineation but shows suboptimal contrast. **b** Subtraction iodine-enhanced image clearly demonstrates extranodal extension into the surrounding fat tissue. **c** MRI short tau inversion recovery sequence shows a positive “flare sign.” **d** Histopathology confirms extension to the perivascular area (v) with associated fibrosis (*), consistent with major pathologic ENE. T: tumor; N: lymph node. Imaging parameters: **a, b** UHR-CT; SHR mode, 1024 × 1024 matrix, 1 mm slice thickness, DLR. **c** 3 T MRI; coronal STIR sequence, 3 mm slice thickness

An accurate diagnosis of iENE Grade 1 requires a detailed evaluation of nodal margins and perinodal fat planes using established imaging criteria (Table 1). Figures 6 and 7 demonstrate cases of Grade 1 iENE, characterized by poorly defined margins, irregularity of the nodal capsule, and obliteration of adjacent fat planes [12, 27, 48–50]. Both cases exhibit the flare sign on MRI—high signal intensity in the interstitial tissues surrounding and extending from the metastatic node on fat-suppressed T2-weighted images—which is considered a highly specific indicator of ENE [51]. Conventional CT/MRI findings have demonstrated a sensitivity of 50%–70% and a specificity exceeding 90% for pENE [21, 48, 51–53]. These diagnostic performance metrics were largely based on earlier studies using pENE as the reference standard. The HNCIG consensus has since refined the diagnostic criteria by excluding capsular thickening and necrosis, which are now classified as Grade 0 iENE [11]. While specific signs such as the flare sign on MRI show promise for increasing diagnostic confidence in assessing Grade 1 iENE, further validation studies are needed to establish their clinical utility in the context of the updated HNCIG criteria.

**Fig. 8:**
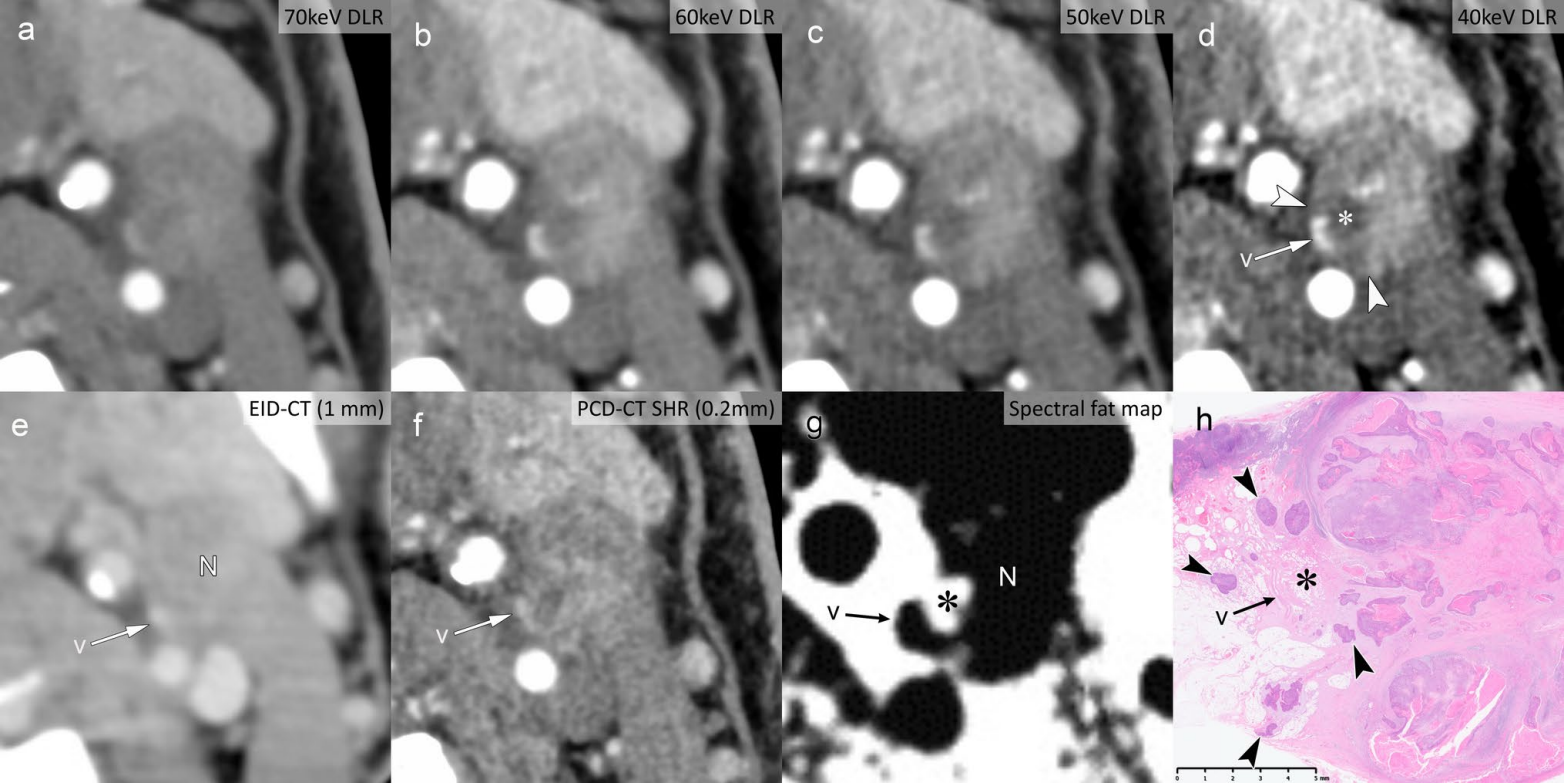
Spectral imaging capabilities of photon-counting detector CT (PCD-CT) demonstrating iENE Grade 1 in a 72 year-old man with hypopharyngeal squamous cell carcinoma (**a-d**). Virtual monochromatic images with a DLR algorithm adapted for PCD-CT show progressive contrast enhancement from 70 to 40 keV. The 40 keV image **d** provides superior soft tissue contrast, clearly delineating small vessels (v) and adjacent low-attenuation areas (*) suggestive of extranodal extension (arrowheads). **e** Conventional EID-CT shows poor spatial resolution, limiting nodal evaluation. **f** PCD-CT super-high resolution demonstrates improved sharpness but limited soft tissue contrast. **g** Spectral fat map confirms that the low-attenuation areas adjacent to vessels in the 40 keV image represent fat tissue, indicating extranodal extension into perinodal fat. **h** Histopathology confirms tumor cell infiltration (arrowheads) around small vessels (v) and into surrounding fat tissue (*), validating major pathological ENE. N: lymph node. Imaging parameters: **a–d, g** PCD-CT; 1024 × 1024 matrix, 1 mm slice thickness, DLR; spectral analysis for VMI (70/60/50/40 keV) and fat map. **e** Conventional EID-CT; 512 × 512 matrix, 3 mm slice thickness. **f** PCD-CT; SHR mode, 1024 × 1024 matrix, 0.2-mm slice thickness, DLR

The development of high-resolution imaging technologies, particularly PCD-CT, has introduced promising advances in detecting Grade 1 iENE. The SHR mode with 1024-matrix capability offers exceptional spatial resolution, as demonstrated in Figs. 6 and 7, where subtle perinodal fat infiltration is more clearly visualized. Figure 8 illustrates how virtual monochromatic imaging at low energy levels (40–50 keV) enhances superior soft tissue contrast, while material decomposition maps facilitate detection of fat plane obliteration—key features of Grade 1 iENE. These technical advances could potentially improve diagnostic accuracy compared to conventional imaging. However, further validation studies comparing PCD-CT performance with conventional imaging and histopathological correlation are needed to establish clinical utility.

## iENE-positive: Grade 2

### Definition

iENE Grade 2 is defined as clear invasion across two or more inseparable adjoining lymph nodes, forming a conglomerate, matted, or coalescent mass, with or without accompanying Grade 1 features **(**Fig. 1**)**. This grade represents a more advanced form of ENE, in which the tumor has not only penetrated individual nodal capsules but has also spread between nodes to create confluent masses through intercapsular invasion. Diagnosis requires unequivocal imaging evidence of true nodal fusion rather than mere anatomical proximity, which helps distinguish actual coalescence from closely situated but separate metastatic nodes.

### Clinical significance

Grade 2 iENE is considered an intermediate yet clinically meaningful adverse prognostic factor that can substantially influence treatment planning in head and neck cancer. Although data are limited, existing studies suggest that coalescent nodal disease may be associated with an increased risk of distant metastasis and poorer overall outcomes compared with isolated nodal metastases [29, 31, 32]. Some evidence also points to a correlation between nodal matting patterns and more aggressive tumor behavior, particularly in nasopharyngeal carcinoma [29, 32]. However, the independent prognostic value of Grade 2 iENE—distinct from Grades 1 and 3—remains to be confirmed through large-scale, prospective investigations. Therefore, although Grade 2 provides a distinct morphological category, its precise impact on patient outcomes and treatment decisions, separate from Grades 1 and 3, is a key area for future investigation.

### Imaging findings for iENE Grade 2 and advantages of PCD-CT

**Fig. 9:**
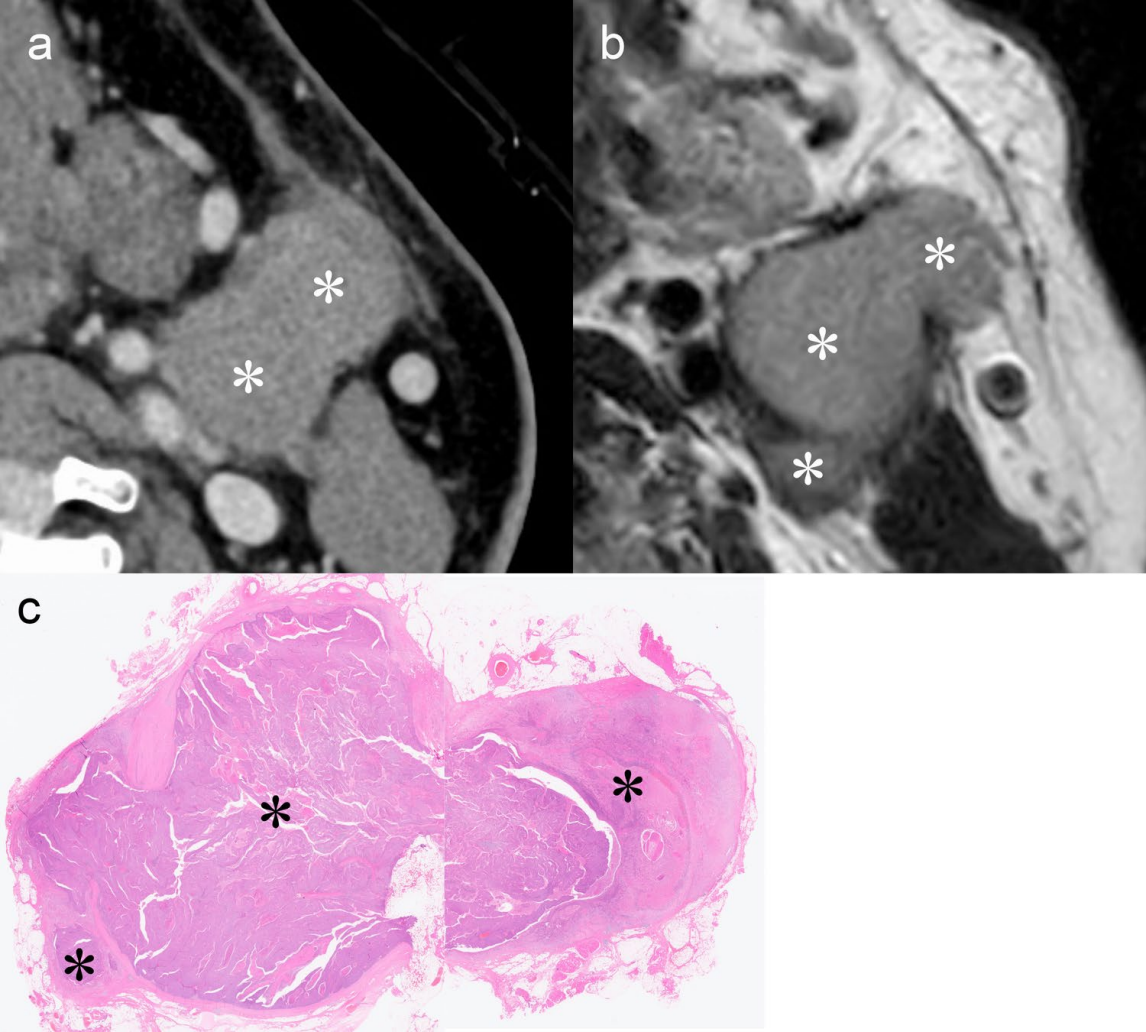
iENE Grade 2 in a 54 year-old man with p16-positive oropharyngeal carcinoma. **a** Ultra-high-resolution CT axial images show multiple lymph node metastases forming a single mass (*). **b** T2-weighted MRI shows fusion of lymph node metastases without clear capsular boundaries (*). **c** Histopathology after neck dissection confirms major pathologic ENE with fusion of multiple metastatic nodes (*). Imaging parameters: **a** UHR-CT; SHR mode, 1024 × 1024 matrix, 1 mm slice thickness, DLR. **b** 3 T MRI; axial T2-weighted sequence, 3 mm slice thickness

**Fig. 10:**
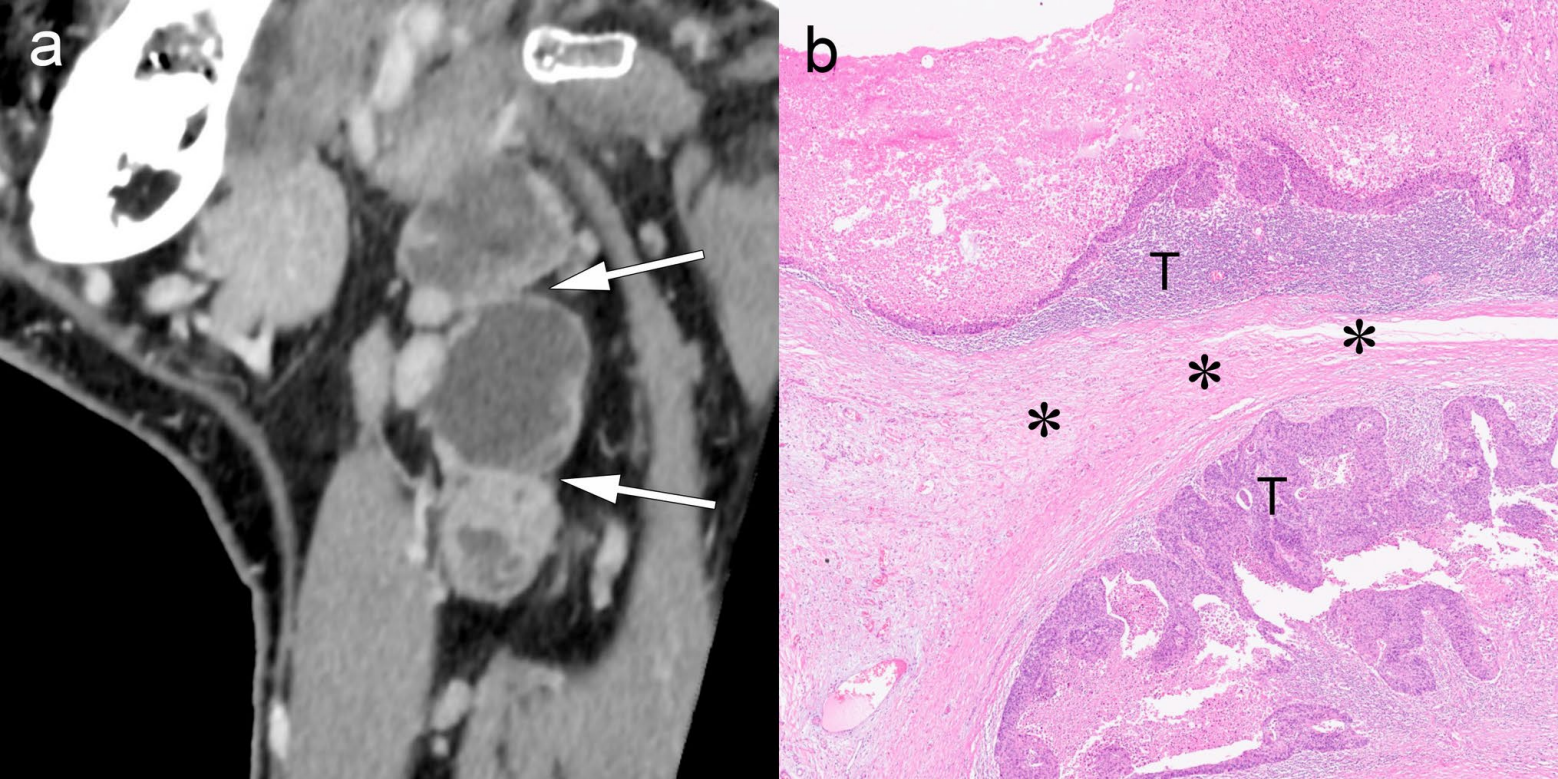
Closely adjacent nodes mimicking iENE Grade 2 (iENE Grade 0). This pENE-negative case serves as a contrast to the true nodal fusion with ENE shown in Fig. 9. A 54 year-old man with hypopharyngeal squamous cell carcinoma. **a** Ultra-high-resolution sagittal images show three lymph node metastases (N) with ill-defined margins (arrowheads) that appear to be in contact; however, clear triangular fat tissue is preserved between the nodes (arrows). **b** Histopathology reveals that the lymph nodes containing metastatic tumor (T) are fused by capsular fibrosis (*) rather than by extranodal extension, confirming the absence of ENE. Imaging parameters: (**a**) UHR-CT; SHR mode, 1024 × 1024 matrix, 1 mm slice thickness, DLR

Radiological assessment of Grade 2 iENE requires careful evaluation to distinguish true coalescence from simple anatomical proximity (Table 1). Figure 9 demonstrates a case of Grade 2, where multiple metastatic nodes have fused into a single conglomerate mass with loss of clear capsular boundaries. Figure 10, by contrast, presents a diagnostic pitfall: multiple nodes that are in close contact but maintain intervening fat planes and capsular integrity, a finding that does not qualify as Grade 2. High-resolution imaging, particularly PCD-CT with ultra-high-resolution capabilities, may enhance diagnostic confidence through improved visualization of individual nodal margins and capsular integrity. The superior spatial resolution enables detailed assessment of each lymph node within a conglomerate mass, potentially allowing better differentiation between genuinely merged nodes versus those in close anatomical contact.

It is important to note that pathological criteria for nodal coalescence differ from radiological iENE Grade 2 criteria. According to the HNCIG pENE consensus, pathological fusion of nodes without extracapsular spread is not considered positive [11, 20]. Further research is needed to evaluate the specific prognostic impact of iENE Grade 2 across different head and neck cancer subsites and to reconcile these criteria across imaging and pathology.

## iENE-positive: Grade 3

### Definition

Grade 3 iENE is defined as clear radiologic evidence of tumor extension into adjacent structures such as muscles, skin, glands, or the neurovascular bundle, with or without accompanying Grade 1 or 2 features **(**Fig. 1**)** [11]. This represents the most advanced form of ENE, in which the tumor infiltrates beyond the perinodal fat to invade critical anatomical structures. Diagnosis requires unequivocal imaging evidence of invasion into specific adjacent structures, distinguishing it from mere contact or mass effect. Grade 3 encompasses a broad spectrum of findings, ranging from muscle involvement to life-threatening invasion of major vessels or other vital structures.

### Clinical significance

iENE Grade 3 carries the most severe prognostic implications of all iENE grades and significantly impacts treatment strategies for head and neck cancer. Importantly, it has been adopted as an N3 criterion in the upcoming AJCC/UICC 9th edition staging system for nasopharyngeal carcinoma, based on full expert panel consensus [15, 16]. In addition, the 9th edition now accepts unequivocal imaging findings as confirmation of cENE, with Grade 3 iENE resulting in upstaging to the highest N categories—N2 for salivary gland carcinoma, N3 for HPV-associated oropharyngeal carcinoma, and N3b for most other head and neck cancer subsites [15]. This marks a shift from the 8th edition, which did not consider radiological evidence as sufficient for diagnosing cENE [8, 27]. This update underscores the growing reliance on radiographic imaging as a standard tool for identifying unequivocal ENEs and establishes Grade 3 iENE as a key criterion in clinical staging and treatment planning.

The specific adjacent structures involved play a crucial role in guiding prognosis and therapeutic decisions. Invasion of the carotid artery—particularly the internal (ICA) or common carotid artery (CCA)—is associated with poor outcomes [54–57] and often defines unresectability [55, 57, 58]. Neurovascular invasion affecting major nerves can lead to denervation changes that are apparent on imaging and physical examination, with subsequent muscle atrophy and functional deficits [59–61]. Skin invasion may necessitate extensive reconstructive procedures, including flap procedures, while muscle invasion can compromise functional outcomes and limit options for organ-preserving treatment strategies [60].

### Imaging findings for iENE Grade 3 and advantages of PCD-CT

**Fig. 11:**
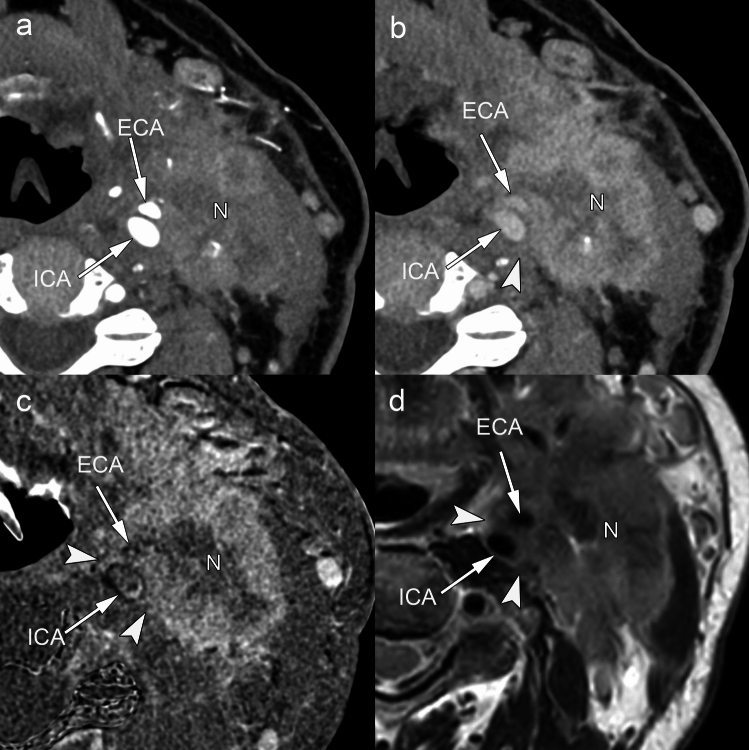
iENE Grade 3 with carotid artery invasion in a 74 year-old man with hypopharyngeal carcinoma. **a** Ultra-high-resolution early phase and **b** delayed phase show tumor extension to the left carotid bifurcation with near-complete encirclement of the external carotid artery (ECA). **c** Ultra-high-resolution subtraction iodine image clearly delineates the extent of tumor invasion. **d** T2-weighted MRI (3 mm slice thickness) confirms ECA encirclement by tumor more than 270°, meeting the criteria for probable carotid invasion and resulting in treatment modification with induction chemotherapy. ICA: internal carotid artery. Imaging parameters: **a-c** UHR-CT; SHR mode, 1024 × 1024 matrix, 1 mm slice thickness, DLR. **d** 3 T MRI; axial T2-weighted sequence, 3 mm slice thickness

**Fig. 12:**
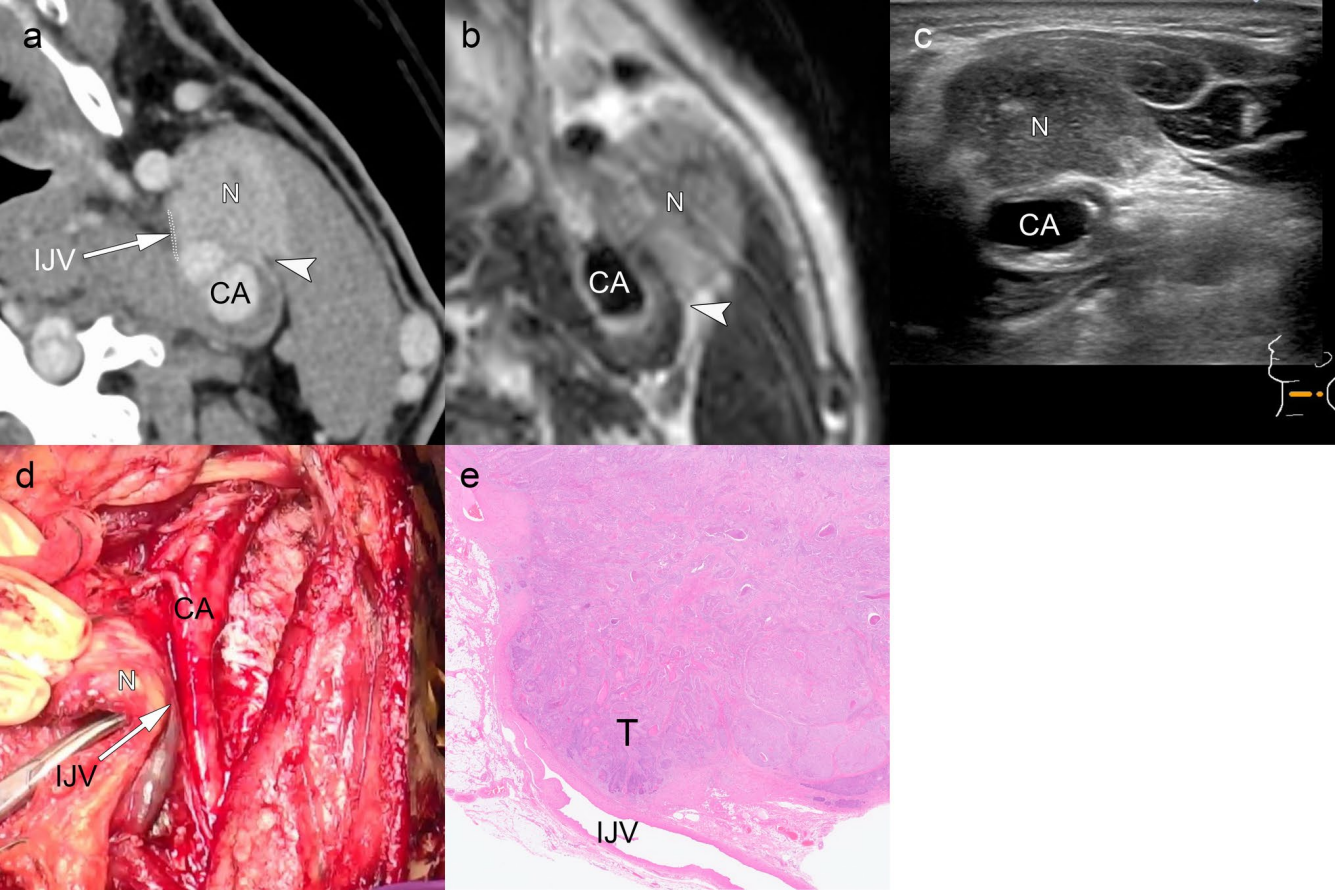
iENE Grade 3 with internal jugular vein invasion in a 66 year-old man with hypopharyngeal squamous cell carcinoma. **a** Ultra-high-resolution image shows lymph node metastasis (N) adjacent to the left carotid bifurcation with irregular margins, suggesting ENE. The left internal jugular vein (IJV) is nearly obliterated, indicating invasion (arrow). Tumor contacts the carotid artery at approximately 90° without encirclement (arrowhead). **b** T2-weighted MRI confirms 90° contact with the carotid artery (arrowhead). **c** Ultrasound demonstrates good mobility between the tumor and carotid artery, excluding carotid invasion (see Supplementary Video 1 for dynamic evaluation). **d** Intraoperative findings show fixed adherence between the lymph node metastasis (N) and IJV (arrow) with dissection from carotid artery possible. **e** Histopathology confirms lymph node metastasis with extranodal extension invading the IJV, consistent with major pathologic ENE. CA: carotid artery. T: tumor; N: lymph node. Imaging parameters: **a** UHR-CT; SHR mode, 1024 × 1024 matrix, 1-mm slice thickness, DLR. **b** 3 T MRI; axial T2-weighted sequence, 3 mm slice thickness

The imaging assessment of Grade 3 iENE requires systematic evaluation of multiple adjacent structures, each presenting unique diagnostic challenges and established criteria (Table 1). For carotid involvement, three key CT/MRI signs have been established as reliable indicators of invasion: (i) > 270° circumferential contact, (ii) focal loss of the pericarotid fat/adventitial plane, and (iii) luminal narrowing or deformation. Arterial involvement specifically requires encasement of major arteries, including the common carotid, internal carotid, external carotid origin, and vertebral arteries; smaller arteries are not included in the assessment [34]. Sign (i) yields 92–100% sensitivity and 88–97% specificity; < 180° contact typically indicates absence of invasion, whereas 180–270° is indeterminate and warrants multidisciplinary review. Fat-plane loss shows 83–91% sensitivity and 80–95% specificity, while luminal change is highly specific (~ 100%) but relatively insensitive (35–70%) [62–65]. Figure 11 demonstrates a case with near-complete encirclement (> 270°) of the external carotid artery at the bifurcation, where PCD-CT with iodine-subtraction imaging clearly delineated the extent of tumor invasion, resulting in treatment modification. Involvement of the carotid bifurcation is considered unresectable regardless of contact angle because surgical access for vascular control is compromised [58, 61, 62]. Contrast-enhanced CT, while useful for triage, tends to overestimate invasion, whereas T2-weighted MRI offers higher specificity, particularly in cases of 180–270° contact [58, 61, 62]. Figure 12 illustrates the importance of multimodal assessment: while this case showed internal jugular vein obliteration confirming venous invasion, the carotid artery had only 90° contact. Real-time ultrasonography confirmed preserved carotid mobility, excluding arterial invasion **(**Fig. 12c**, **Supplementary Video 1**)** [66]. This case emphasizes that for internal jugular vein invasion, compression or displacement alone is insufficient; tumor thrombus within the vein or complete obliteration without flow must be demonstrated [34].

**Fig. 13:**
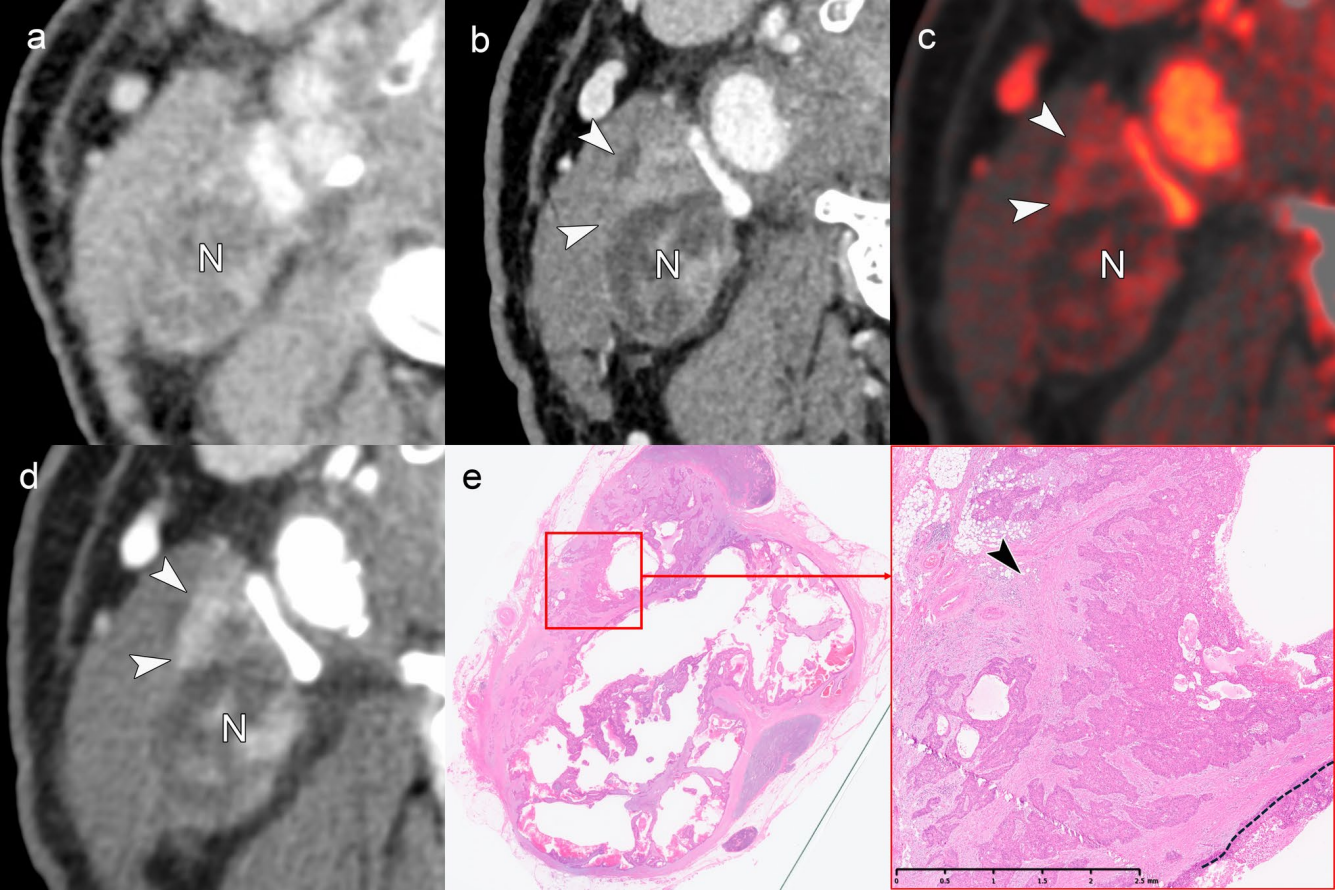
PCD-CT spectral imaging for sternocleidomastoid muscle invasion assessment in iENE Grade 1. An 81 year-old man with hypopharyngeal SCC and right level IIA lymph node metastasis. **a** Conventional CT shows limited assessment of nodal margins. **b** PCD-CT ultra-high-resolution image demonstrates extranodal extension (arrowheads) but offers suboptimal evaluation of the sternocleidomastoid muscle interface. **c** Spectral iodine image enhances visualization of the lymph node-muscle boundary (arrowheads). **d** Virtual monochromatic imaging at 40 keV provides superior soft tissue contrast, clearly delineating the intact sternocleidomastoid muscle interface (arrowheads), confirming the absence of muscle invasion. **e** Histopathology reveals major pathologic extranodal extension (black arrowhead) without muscle invasion, correlating with intraoperative findings that confirmed preservation of the muscle plane. N: lymph node. Imaging parameters: **a** conventional EID-CT; 1-mm slice thickness. **b** PCD-CT; SHR mode, 1024 × 1024 matrix, 0.2 mm slice thickness, DLR. **c, d** PCD-CT; 512 × 512 matrix, 1 mm slice thickness, DLR; spectral analysis for iodine image (**c**) and VMI 40 keV (**d**)

MRI is the preferred first-line modality for assessing nonvascular Grade 3 iENE targets—such as muscles, bones, nerves, major glands, and skin—due to its superior soft tissue contrast. Muscle invasion (Grade 3) is suspected when ENE obliterates the fascial plane and directly involves the sternocleidomastoid or strap muscles [27, 50]. Although both CT and MRI can detect frank muscle fiber disruption, strict criteria—such as unequivocal infiltration in at least two contiguous sections—are essential to avoid misinterpreting subtle stranding. Differentiating perinodal fat extension (Grade 1) from muscle/bone invasion (Grade 3) is crucial, as the latter determines the extent of resection required. Advanced CT techniques including spectral imaging can assist in challenging cases [17, 67–72]. Figure 13 illustrates the value of PCD-CT spectral imaging in excluding muscle invasion: while ultra-high-resolution imaging confirmed ENE, spectral iodine maps and virtual monochromatic imaging at 40 keV provided enhanced soft tissue contrast that clearly demonstrated an intact sternocleidomastoid muscle interface, thereby excluding Grade 3 muscle invasion and confirming Grade 1 iENE [70, 71].

**Fig. 14:**
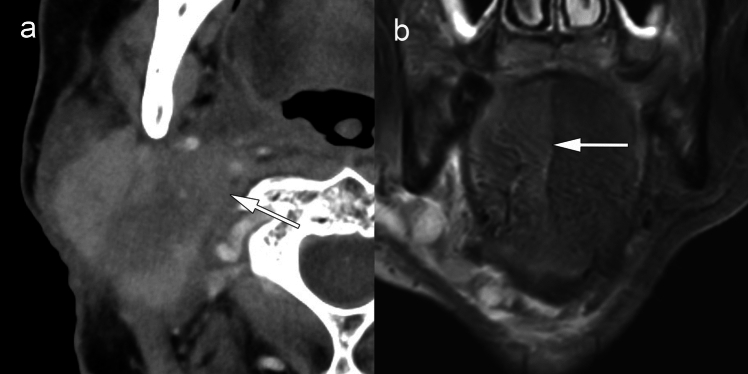
iENE Grade 3 with hypoglossal nerve invasion in a 64 year-old man with recurrent lymph node metastasis. **a** Contrast-enhanced CT shows level IIA extranodal extension invading the area around the posterior belly of the digastric muscle (arrow). **b** MRI short tau inversion recovery coronal image reveals right-sided tongue edema (arrows), indicating hypoglossal nerve paralysis due to extranodal extension. Imaging parameters: **a** conventional EID-CT; 512 × 512 matrix, 1 mm slice thickness. **b** 3 T MRI; coronal STIR sequence, 3 mm slice thickness

**Fig. 15:**
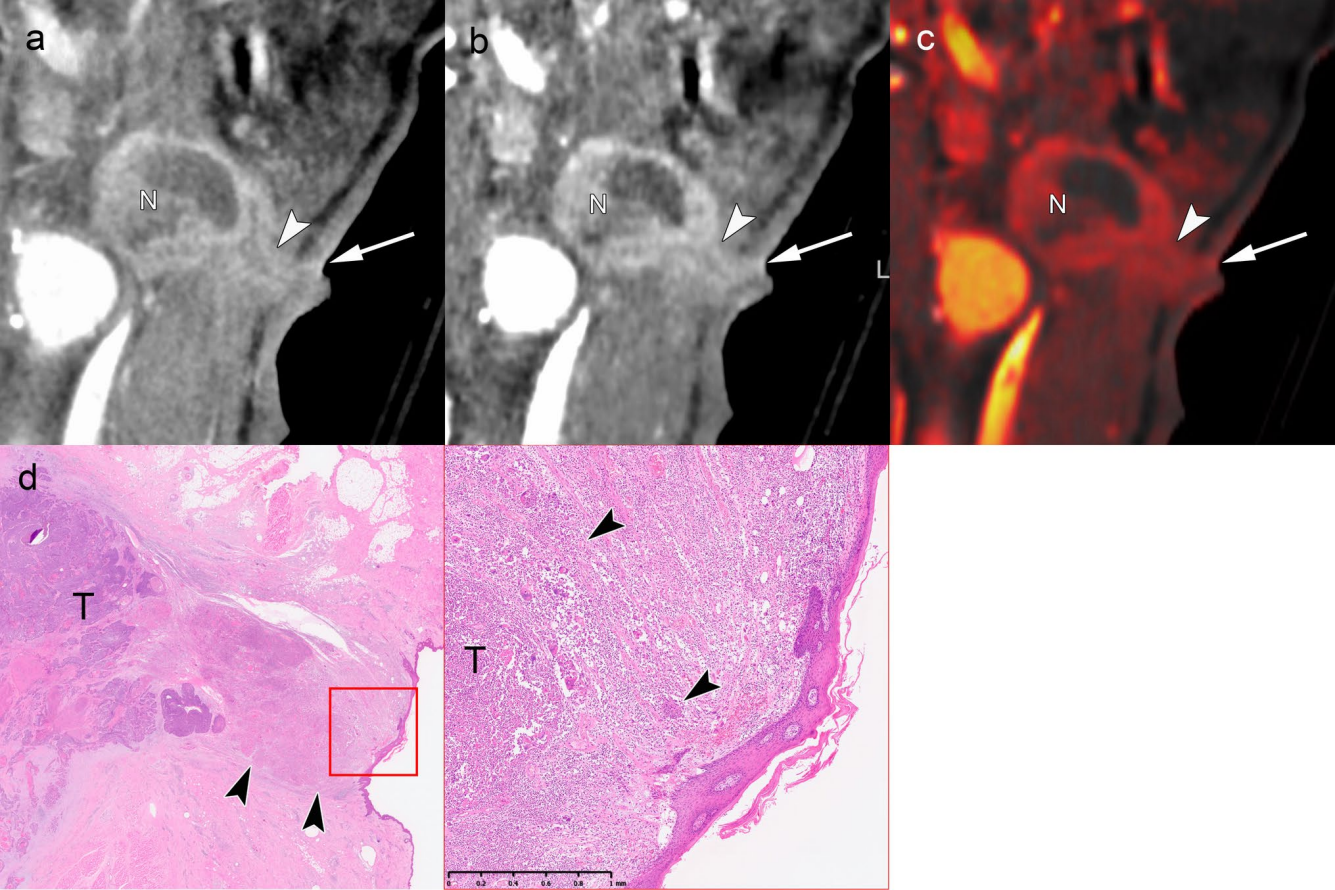
iENE Grade 3 with skin invasion in an 80 year-old man with hypopharyngeal squamous cell carcinoma. **a** PCD-CT ultra-high-resolution coronal image shows soft tissue thickening extending from the left level IIA lymph node metastasis to the skin (arrow), with indistinct subcutaneous fat (arrowhead). **b, c** PCD-CT spectral images (40 keV and iodine map) clearly demonstrate tumor extension to the skin (arrowhead, arrow). **d** Histopathological findings after neck dissection with skin resection confirm squamous cell carcinoma invasion into the dermis (arrowheads), consistent with major pathologic ENE. T: tumor; N: lymph node. Imaging parameters: **a** PCD-CT; SHR mode, 1024 × 1024 matrix, 0.5 mm slice thickness, DLR. **b, c** PCD-CT; 512 × 512 matrix, 1 mm slice thickness, DLR; spectral analysis for VMI 40 keV (**b**) and iodine map (**c**)

Nerve invasion related to nodal ENE, distinct from classical perineural spread, is suggested when a metastatic node effaces the perineural fat plane and is accompanied by nerve thickening or signs of denervation. Figure 14 shows hypoglossal nerve invasion with associated tongue edema indicating denervation [27, 59]. Cutaneous infiltration, though rare, is associated with poor prognosis and may present with loss of the subcutaneous fat stripe, focal skin thickening, or ulceration adjacent to the node. Figure 15 demonstrates skin invasion where PCD-CT spectral imaging clearly delineated tumor extension through the subcutaneous fat to the dermis. For major glands, definitive invasion through the capsule into the parenchyma is required; displacement or distortion alone is insufficient [34].

Advanced imaging technologies—particularly PCD-CT with 1024-matrix capability and spectral imaging features—offer potential advantages for Grade 3 iENE assessment through improved spatial resolution and tissue characterization. These capabilities may enhance diagnostic confidence in complex cases, though additional validation studies are required to establish their clinical impact.

## Future challenges and implementation

Key challenges moving forward include validating inter-observer agreement using the HNCIG consensus criteria across institutions and among readers with varying levels of experience. Large-scale studies are needed to confirm the prognostic significance of each iENE grade across different head and neck cancer subsites and to clarify how the new grading system—particularly Grade 2 (coalescent nodes)—enhances risk stratification. Furthermore, it is important to investigate whether advanced techniques, such as PCD-CT spectral capabilities including virtual monochromatic imaging and iodine map, can enhance the detection of subtle Grade 1 iENE, and further studies are needed to directly compare the diagnostic performance of PCD-CT with conventional CT and MRI. Standardizing imaging protocols for these technologies will help ensure consistent iENE evaluation and facilitate their incorporation into future clinical trials.

## Conclusion

iENE has become an essential and objective prognostic indicator in head and neck cancer, now formally recognized in the UICC 9th edition staging system. However, iENE diagnosis remains inherently subjective, and inter-observer and inter-institutional variability will persist until standardized diagnostic criteria are widely implemented. At present, there are no universally accepted recommendations regarding specific imaging modalities or reconstruction protocols for iENE assessment.

The primary aim of iENE assessment is to enhance patient-specific risk stratification and guide treatment intensity decisions. The HNCIG consensus criteria offer a structured framework for this assessment, while advanced imaging technologies, such as PCD-CT, offer new possibilities for improved diagnostic accuracy. Radiologists are encouraged to support the appropriate application of these tools and standardized criteria to reduce diagnostic uncertainty. Through more precise iENE assessment and staging, we can improve patient outcomes and support personalized approaches to head and neck cancer care.

## Supplementary Information

Below is the link to the electronic supplementary material.

Supplementary file1 (MP4 14151 KB)
